# Exosomal miR‐6733‐5p mediates cross‐talk between glioblastoma stem cells and macrophages and promotes glioblastoma multiform progression synergistically

**DOI:** 10.1111/cns.14296

**Published:** 2023-06-12

**Authors:** Shilu Huang, Liang Liu, Zhipeng Xu, Xinglei Liu, Anyi Wu, Xiaopei Zhang, Zengyang Li, Suwen Li, Yongdong Li, Jiaqi Yuan, Shan Cheng, Haoran Li, Jun Dong

**Affiliations:** ^1^ Department of Neurosurgery Second Affiliated Hospital of Soochow University Suzhou China; ^2^ Department of Neurosurgery Affiliated Nanjing Brain Hospital, Nanjing Medical University Nanjing China

**Keywords:** exosomes, glioblastoma stem cells, macrophage polarization, microRNA‐6733‐5p, stemness

## Abstract

**Aim:**

Exosomal miRNAs derived from glioblastoma stem cells (GSCs) are important mediators of immunosuppressive microenvironment formation in glioblastoma multiform (GBM), especially in M2‐like polarization of tumor‐associated macrophages (TAMs). However, the exact mechanisms by which GSCs‐derived exosomes (GSCs‐exo) facilitate the remodeling of the immunosuppressive microenvironment of GBM have not been elucidated.

**Methods:**

Transmission electron microscopy (TME) and nanoparticle tracking analysis (NTA) were applied to verify the existence of GSCs‐derived exosomes. Sphere formation assays, flow cytometry, and tumor xenograft transplantation assays were performed to identify the exact roles of exosomal miR‐6733‐5p. Then, the mechanisms of miR‐6733‐5p and its downstream target gene regulating crosstalk between GSCs cells and M2 macrophages were further investigated.

**Results:**

GSCs‐derived exosomal miR‐6733‐5p induce macrophage M2 polarization of TAMs by positively targeting IGF2BP3 to activate the AKT signaling pathway, which further facilitates the self‐renewal and stemness of GSCs.

**Conclusion:**

GSCs secrete miR‐6733‐5p‐rich exosomes to induce M2‐like polarization of macrophages, as well as enhance GSCs stemness and promote malignant behaviors of GBM through IGF2BP3 activated AKT pathway. Targeting GSCs exosomal miR‐6733‐5p may provide a potential new strategy against GBM.

## Graphical abstract

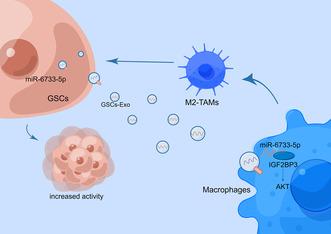

## 1 INTRODUCTION

Glioblastoma multiforme (GBM) accounts for 82% of malignant brain tumors and actually remains the most common and lethal form of cancer in the central nervous system.^1^ GBM are prone to recurrence and the 5‐year survival rate is less than 5%, even after surgery, chemotherapy, radiation, and immunotherapy.^2^ Glioblastoma stem cells (GSCs) contribute to treatment resistance and have the ability to repopulate the tumor causing recurrence.^3^ Hence, an understanding of the specific functions of GSCs as well as the mechanisms of remodeling tumor microenvironment will help to develop a more effective therapeutic strategy.

Tumor‐associated macrophages (TAMs), play vital roles in constructing immuno‐suppressive tumor microenvironment, are enriched in GBMs, and promote tumor growth.^4^ High TAM infiltration has been shown to predict a poor prognosis in variety of malignancies, including GBMs.^5^, ^6^ Several markers such as MHCII, CD80, and iNOS have been identified as M1 subtype TAM that can prevent tumor progression, while other markers including CD163, IL‐10, and Arg1 are regarded as M2 subtype TAM that can promote GBM progression.^7^ Recent studies suggested that both TAMs and GSCs are localized and maintained in tumor perivascular niches,^8^ and GSCs play a critical role in promoting TAM recruitment resulting in GBM development.^9^ Moreover, M2 polarization of TAMs and tissue remodeling of GSCs have been reported to be increased in recurrent GBMs after treatment.^10^ The cross‐talking between TAMs and GSCs plays an important role in the constructing of highly immunosuppressive GBM microenvironment.^11^ However, the underlying molecular mechanisms between their cross‐talk have not yet been fully elucidated.

Exosomes are nanovesicles (30–100 nm in diameter) that originate from the endosomes that can deliver their cargos including RNAs, DNAs, and proteins to recipient cells for intercellular communications.^12^, ^13^, ^14^ Exosomal miRNAs from tumor cells have been found to regulate essential aspects of cancer, including angiogenesis, immunosuppression, and metastasis by remodeling the phenotype and function of recipient cells.^15^, ^16^ Existing research suggests that miRNAs can enhance their downstream target gene expression by binding to the sequences in their promoter, enhancer, 3′‐untranslated region (UTR), or 5′‐UTR.^17^, ^18^, ^19^ However, GSCs exosomal miRNAs mediated remodeling on TAMs, and the relevant regulation mechanism have not been extensively investigated.

In the current studies, we investigated the mutual interactions between GSCs and macrophages by co‐culture transwell system and further evaluated whether GSC‐derived exosomes (GSCs‐exo) induce M2 macrophage polarization via secretion of specific miRNA, which may serve as a potential novel target against GBMs.

## 2 METHODS

### 2.1 Clinical specimens

Clinical GBM tissue specimens were collected from patients who underwent surgical removal and were diagnosed with GBM according to World Health Organization (WHO) pathological criteria at the Department of Neurosurgery, the Second Affiliated Hospital of Soochow University. The pathological diagnosis of GBM was independently achieved by two senior and experienced pathologists. Ethical approval was obtained from the Second Affiliated Hospital of Soochow University (Approval number 2022148). Informed consent was obtained from all subjects.

### 2.2 Cell lines and cell culture

Human glioma stem cell lines GSC11 and GSC23 (M.D. Anderson Cancer Center) were cultured in Dulbecco's modified Eagle's medium (DMEM)/F12 medium (Gibco) containing 20 ng/mL basic fibroblast growth factor (bFGF) (Gibco) and 20 ng/mL epidermal growth factor (EGF) (Gibco). THP‐1 cell line (ATCC) was cultured in RPMI 1640 (Gibco) medium supplemented with 10% heat‐inactivated fetal bovine serum (FBS; Invitrogen), then incubated with 100 ng/mL phorbol‐12‐myristate‐13‐acetate (PMA; Sigma–Aldrich) for 24–48 h to induce resting macrophages (M0). Primary monocyte‐derived macrophages were prepared from fresh blood from healthy volunteers. The study was approved by the Research Ethics Committee of the Second Affiliated Hospital of Soochow University, and written informed consent was obtained from all participants. Peripheral blood mononuclear cells (PBMCs) were isolated via density gradient centrifugation using Ficoll‐Paque (TBDScience LTS1077) at 2000 *g* for 25 min using minimum acceleration and no brake. PBMC fractions were washed in sterile PBS after lysing erythrocytes (CWBIO, CW0613) and plated to select for adherent cells. Non‐adherent cells were washed away after 6 h and the remaining cells incubated in RPMI 1640 medium (Gibco) supplemented with 10% heat‐inactivated pooled human serum (Sigma) and 40 ng/mL macrophage colony‐stimulating factor (M‐CSF, PeproTech, 300‐25). Medium changed every 3 days until the cells differentiated into macrophages by 7 days. All cells were maintained in a humidified chamber containing 5% CO_2_ at 37°C.

### 2.3 Cell transfection

The miR‐6733‐5p mimics/inhibitors and the IGF2BP3 small interfering RNAs (GenePharma, Shanghai, China) were transiently transfected using siRNA‐mate (GenePharma) according to the manufacturer's instructions. Sequences information is shown in Table S2. Plasmid vector overexpressed IGF2BP3 (FuBio) was transfected using Lipofectamine 3000 (Invitrogen) following the manufacturer's instructions.

### 2.4 RT‐qPCR

Total RNA was extracted from cells and tissues with TRIzol (Yeasen). The miRNAs were reversed transcribed using miRNA‐specific stem‐loop RT primers using the RevertAid First Strand cDNA Synthesis Kit (ThermoFisher) and mRNA were transcribed into cDNA using the NovoScript Plus All‐in‐one 1st Strand cDNA Synthesis SuperMix (gDNA Purge; Novoprotein), respectively, according to the manufacturer's protocol. The above cDNAs were then amplified using the NovoStart SYBR qPCR SuperMix Plus Kit (Novoprotein). RT‐qPCR was performed with 10‐μL reaction mixtures containing 5‐μL 2× NovoStart SYBR qPCR SuperMix Plus Kit (Novoprotein), 0.5‐μL forward primer (10 μM), 0.5‐μL reverse primer (10 μM), 1‐μL total DNA (100 ng/μL), and 3‐μL RNase‐free ddH_2_O. The relative expression of miRNA and mRNA was normalized to U6 and GAPDH, respectively. Primer information is shown in Table S1. The final data were analyzed using the $2^{-\Delta \Delta C_{t}}$ method. The RT‐qPCR assay was performed with three technical replicates, and at least three independent biological replicates were performed and showed similar results.

### 2.5 Western blot

Cells or exosomes were lysed in radioimmune precipitation assay (RIPA) lysis buffer (Beyotime) containing phosphatase and protease inhibitor cocktails (Abcam). A total of 20–30 μg of protein sample was subjected to SDS‐PAGE gel, then transferred onto 0.22‐mm polyvinylidene difluoride (PVDF) membranes (Millipore). Membrane was blocked in 5% nonfat milk and incubated with the corresponding primary antibodies at 4°C for overnight, following by incubation with the horseradish peroxidase‐conjugated (HRP) secondary antibody for 2 h at room temperature. Finally, the membrane was detected using FDbio‐Femto ECL (Fudebio) and a chemiluminescence system (Bio‐Rad). The primary antibodies for Western blot were anti‐CD9 (1:1000; abcam, ab263019), anti‐TSG101 (1:1000; abcam, 125011), anti‐HSP70 (1:1000; abcam, ab125011), anti‐Calnexin (1:1000; abcam, ab133615), anti‐IMP3 (1:1000; abcam, ab179807), anti‐pan‐AKT (1:1000; abcam, ab8805), anti‐AKT (1:1000; abcam, ab192623), anti‐GAPDH (1:10,000; abcam, 181602), anti‐OCT4 (1:1000; abcam, ab184665), and anti‐CD133 (1:1000; abclonal, a12711), respectively.

### 2.6 Transwell assay

Transwell inserts (8.0 μm, Corning) were used to evaluate the migration ability of macrophages (THP1 + PMA) cells. The upper chambers contained 300 μL of 2% FBS‐containing RPMI‐1640 medium, while the lower chambers contained 600 μL of 10% FBS‐containing RPMI‐1640 medium. After 48 h, cells in the upper chamber were wiped and the cells that migrated were captured with a microscope (AMG EVOS).

### 2.7 Indirect coculture of macrophages and GSCs

Macrophages were cocultured with GSCs (GSC11 and GSC23) using a six‐well transwell co‐culture system (Corning, Polycarbonate, pore size: 0.4 μm). THP‐1 differentiated macrophages (5 × 10^4^ cells) were seeded in the lower chambers. After 48 h, 5 × 10^4^ cells from GSCs were seeded in the upper chambers. After 48 h of coculture, macrophages or GSCs were, respectively, collected for further analysis.

### 2.8 Isolation and purification of exosomes

The culture supernatant was collected from GSCs (GSC11 or GSC23) culture in DMEM/F12 medium containing bFGF and EGF for 4 days, NHA‐derived exosomes were harvested from NHAs culture medium (DMEM supplemented with 10% exosome‐depleted FBS) for 2 days under 5% CO_2_ at 37°C. Briefly, the collected culture medium was centrifuged at 2000 *g* for 30 min, then 12,000 *g* for 45 min to remove cell debris and large vesicles. For exosome purification, the supernatant was ultracentrifuged at 100,000 *g* for 70 min at 4°C to collect the pellet, then was resuspended in 50–100 μL PBS for the subsequent studies. The concentration of exosomes was detected using a BCA Protein Assay (Beyotime). For exosome addition, the culture medium of recipient cells was supplemented with purified exosomes at 20 μg/mL unless otherwise specified.

### 2.9 Electron microscopy and nanoparticle tracking analysis

Exosomes to be examined by TEM were applied to assess the morphology of exosomes. In brief, exosomes (10 μg) fixed with 4% formaldehyde were placed on copper grids, washed with filtered PBS, and stained with uranyl acetate solution. After 24 h of incubation, samples were analyzed with TEM (Hitachi HT‐7700). Besides, the size distribution and concentration were detected by NTA (ZetaView PMX 110, Particle Metrix).

### 2.10 Engulf of exosomes by macrophages

Purified exosomes were collected and labeled with PKH26 Red Fluorescent membrane linker dye (Sigma‐Aldrich) according to the manufacturer's instructions. THP‐1 cells were seeded in eight‐well chamber slides (5000 cells/well) and pretreated with PMA for 24 h. Then, 10 μg exosomes were incubated with PKH26 dye at room temperature for 5 min. After centrifuged at 10,000 *g* for 30 min at 4°C, the labeled exosome pellets were resuspended and added to THP‐1 derived macrophages for exosomes uptake studies. After incubation for 8 h at 37°C, cells were fixed, stained with DAPI (Invitrogen), and examined by confocal microscope (Zeiss).

### 2.11 ELISA

Cell culture medium was collected 48 h after the indicated treatment. Secretion of IL‐10 and TNF‐α was examined using ELISA Kit (Biolengend, 430607, 430207) according to the manufacturer's instructions.

### 2.12 Flow cytometry

To detect CD11b^+^CD163^+^ macrophages, anti‐CD11b‐FITC (Biolegend, 301330) and anti‐CD163‐PE (Biolegend, 333606) monoclonal antibodies were applied to stain cells. To detect CD133^+^OCT4^+^GSC11 cells, anti‐CD133‐APC (Biolegend, 372805) and anti‐OCT4‐PE (Biolegend, 653703) antibodies were utilized. To detect CD133^+^ GSC23 cells, anti‐CD133‐APC were applied. Cells were harvested, washed, and incubated in blocking buffer. GSC11 cells were permeabilized with 1% Triton X‐100 for 10 min before stained anti‐OCT4‐PE. Flow cytometry was performed using a Cytoflex flow cytometer (Beckman Coulter).

### 2.13 Dual‐luciferase reporter assay

The 293T cells were cotransfected with wild‐type or mutant IGF2BP3 3′/5′‐UTR pGL3 plasmid (FuBio), miR‐6733‐5p mimics or control, and miR‐6733‐5p inhibitors (GenePharma), respectively, with Lipofectamine 3000 (Invitrogen). Luciferase activity was detected using the Dual‐Luciferase Reporter Assay System following the manufacturer's instructions (Promega).

### 2.14 Sphere‐formation assay

GSCs were cocultured with GSC‐exo activated macrophages for 2 days. GSCs were dispersed into single cell suspension and seeded 1000 GSCs into each well of the 96‐well plate with 100 μL DMEM/F12 medium containing bFGF and EGF.

### 2.15 Immunofluorescence (IF), immune‐histochemistry staining (IHC), and hematoxylin–eosin (HE) staining

For the immunofluorescent staining experiment, tumor sections were fixed in 4% paraformaldehyde (PFA) for 30 min, washed three times with PBS, then permeabilized in PBS containing 0.5% Triton X‐100 for 20 min. Samples were blocked with 5% albumin from bovine serum with 0.5% Triton X‐100 in PBS for 1 h at room temperature, then incubated with the primary antibodies against CD163 (1:200, ProteinTech, 16646‐1‐AP) or CD206 (1:100, Zen BioScience, 360017) overnight at 4°C, followed by the corresponding secondary fluorescently labeled antibodies for 1 h at room temperature. The nuclei were counterstained with DAPI (Invitrogen). Images were acquired via confocal microscope (Zeiss).

The brains of xenograft mice were fixed with 4% PFA and embedded in paraffin. Then, 5 μm slices were cut by a microtome (Leica) and deparaffinized, dehydrated, and incubated in heat‐mediated antigen retrieval. Subsequently, the endogenous catalase was eliminated with 3% H_2_O_2_‐methanol, and tissue slices were incubated with indicated primary antibodies against Ki67 (1:500, Servicebio, GB121141), IMP3 (1:500; abcam, ab179807) and F4/80 (1:2000, Sanying, 28463‐1‐AP) at 4°C overnight. After washing with PBS, sections were incubated with biotinylated secondary antibodies at room temperature for 1 h. They were then incubated with peroxidase solution for 30 min and then the sections were stained with DAB reagent and counterstained with hematoxylin. The images of each section were taken and analyzed under an optical microscope.

In HE staining, the paraffin‐embedded sections were sequentially deparaffinized, dehydrated, stained by hematoxylin, differentiated by the addition of hydrochloric ethanol, backed to blue with ammonia water, and stained with eosin. Then, the sections were dehydrated with gradient alcohol, cleared with xylene, and sealed with neutral resin. Finally, tissue HE staining photos were acquired under an optical microscope.

### 2.16 Animal studies

Female BALB/c nude mice (4 weeks old, 15–20 g) were randomly divided into four groups. For the subcutaneous xenografts, 5 × 10^6^ GSC23 cells were mixed with 5 × 10^5^ conditioned macrophages stimulated by PBS, GSCs‐exo, or miR‐6733‐5p mimics transfection, respectively, at a ratio of 10:1 ratio were injected subcutaneously into the right flank of each mouse, respectively. The mice were sacrificed 4 weeks after tumor inoculation. The tumors were obtained by surgery and the tumor volume and weight were measured and calculated.

For the orthotopic xenografts, 5 × 10^5^ GSC23 cells were mixed with 5 × 10^4^ conditioned macrophages stimulated by PBS, GSCs‐exo, or miR‐6733‐5p mimics transfection, respectively, at a ratio of 10:1 ratio orthotopically xenografted by intracerebral injection into the right cerebral cortex of the mouse at a depth of 3.5 mm. Mice were euthanized when neurological symptoms were observed. Then, the whole brain was harvested, PFA‐fixed, paraffin‐embedded, and sectioned coronally from anterior to posterior. All the animal experiments were approved by the Institutional Animal Care and Use Committee of Second Affiliated Hospital of Soochow University.

### 2.17 Statistical analysis

All statistical analysis were conducted with GraphPad Prism 9.0 (GraphPad Software). Results are presented as means ± SD standard deviation on three independent experiments. The normality of the data distribution was analyzed by the Shapiro–Wilk test. Student's *t* test was performed to analyze the statistical difference between two groups, and analysis of variance (ANOVA) was applied to evaluate the differences between multiple groups. The *p*‐value <0.05 was considered statistically significant (*p < 0.05; **p < 0.01; ***p < 0.001; ****p < 0.0001). *p*‐Value >0.05 was considered not significant and was denoted by “NS.”

## 3 RESULTS

### 3.1 TAMs were accumulated in GBM and can be induced transformation toward M2 polarization via GSCs

**FIGURE 1:**
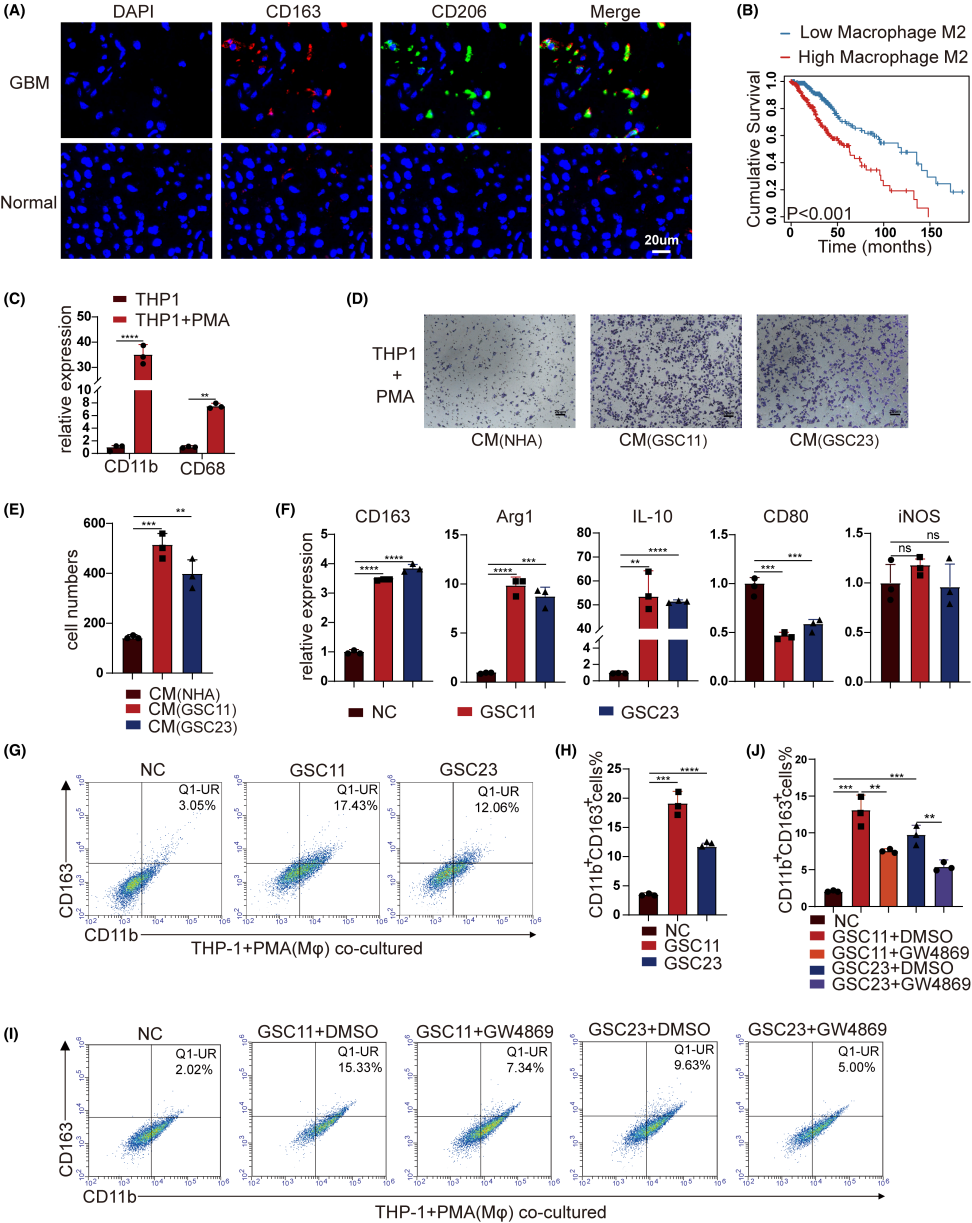
Glioblastoma stem cells (GSCs) induce macrophage toward M2 polarization which infiltrates more in GBM. (A) Representative immunofluorescent (IF) staining of M2‐like TAMs marker (CD163 and CD206) in human GBM tissues. Nuclei were counterstained with DAPI (blue). Scale bar represents 20 μm. (B) Kaplan–Meier survival plots of M2‐like TAMs signature score showed a higher score indicated a poorer prognosis. *p* ˂ 0.001, log‐rank test. (C) The expression levels of M0 markers (CD11b and CD68) were determined by RT‐qPCR. (D, E) Conditioned media of GSC promoted macrophage migration through transwell filters. (F) After PMA induction, THP1 (Mφ) were cocultured with GSCs. The expression levels of M2 markers (CD163, Arg1, and IL10) and M1 markers (CD80 and iNOS) were determined by RT‐qPCR. (G) THP1 (Mφ) were cocultured with GSCs (GSC11 or GSC23). Flow cytometry was applied to measure macrophage marker (CD11b) and M2 macrophage‐related phenotypic marker (CD163) on the surfaces of macrophages. (H) The ratio of CD11b‐positive plus CD163‐positive (CD11b^+^CD163^+^) macrophages were quantitated. (I, J) Macrophages were cocultured with GSCs treated with DMSO or exosome secretion inhibitor GW4869. Flow cytometry and quantification were performed to analyze the proportion of CD11b^+^CD163^+^ macrophages. Data depict the mean ± standard deviation and are representative of three independent experiments (*p < 0.05; **p < 0.01; ***p < 0.001; ****p < 0.0001).

To investigate the population of M2‐like TAMs in surgical specimens of GBM patients, immunofluorescence staining of M2‐like TAMs makers CD163 and CD206 was performed, which disclosed that CD163 and CD206 expression elevated higher in tumor tissue in comparison to para‐tumor tissue (Figure 1A). Further investigations focus on exploring the relationships between M2‐like TAMs signature and clinic prognosis of GBM with Kaplan–Meier analysis, which demonstrated that higher M2‐like TAMs infiltration indicated poorer survival of GBM patients (Figure 1B; Figure S1A). M2‐like TAMs infiltration and accumulation can be observed in GBM, which was in accordance with the previous studies.^5^, ^6^

In vitro differentiation of THP‐1 cells into macrophages was achieved with addition of PMA (100 ng/mL), which was verified by both adherent cells morphological changes (Figure S1B) and increased expression of the cell surface markers CD11b and CD68 (Figure 1C). To explore whether GSCs were involved in promoting the infiltration of M2‐like TAMs in the GBM microenvironment, the key stemness biomarkers were examined (Figure S1C,D) and a coculture model was established based on transwell chambers in vitro. The transwell migration assay showed that conditioned media (CM) of GSC11 and GSC23 promoted the migration of M0 macrophages differentiated by THP‐1, compared to the CM of normal human astrocytes (NHAs) group (Figure 1D,E). To further explore whether GSCs mediated the M2 polarization of macrophages, M0 macrophages derived from THP‐1 cells were cocultured with GSCs in a transwell system (Figure S1E). Two days later, the levels of macrophage‐associated phenotypic markers were detected by RT‐qPCR, the level of M2 macrophages markers (CD163, Arg1, and IL‐10) in macrophages cocultured with GSCs (GSC11 or GSC23 cells) significantly increased, while one of the M1 markers CD80 slightly decreased, and another M1 marker iNOS kept stable, compared with the naive resting macrophages (Mφ) group (Figure 1F). Flow cytometry analysis further verified that the double positive expression rate of the M0 marker CD11b and M2 marker CD163 elevated obviously after mutual interactions with GSC11 or GSC23 cells in vitro (Figure 1G,H). For exosomes are important vehicles for intercellular communications,^20^ whether GSCs exosomes were involved in mediating the M2 polarization of macrophages needs further investigations. Addition of GW4869 resulted in inhibition of GSCs exosomes secretion, RT‐qPCR disclosed the level of M2 markers (CD163, Arg1, and IL‐10) in macrophages decreased simultaneously (Figure S1F,G), flow cytometric analysis showed that the proportion of CD11b^+^CD163^+^ double positive macrophages significantly decreased after inhibition of GSCs exosomes production (Figure 1I,J), which implied the possibility that GSCs induced M2 polarization of macrophages in an exosome‐dependent manner.

### 3.2 GSCs induced M2 polarization of macrophages in exosome‐dependent manner

**FIGURE 2:**
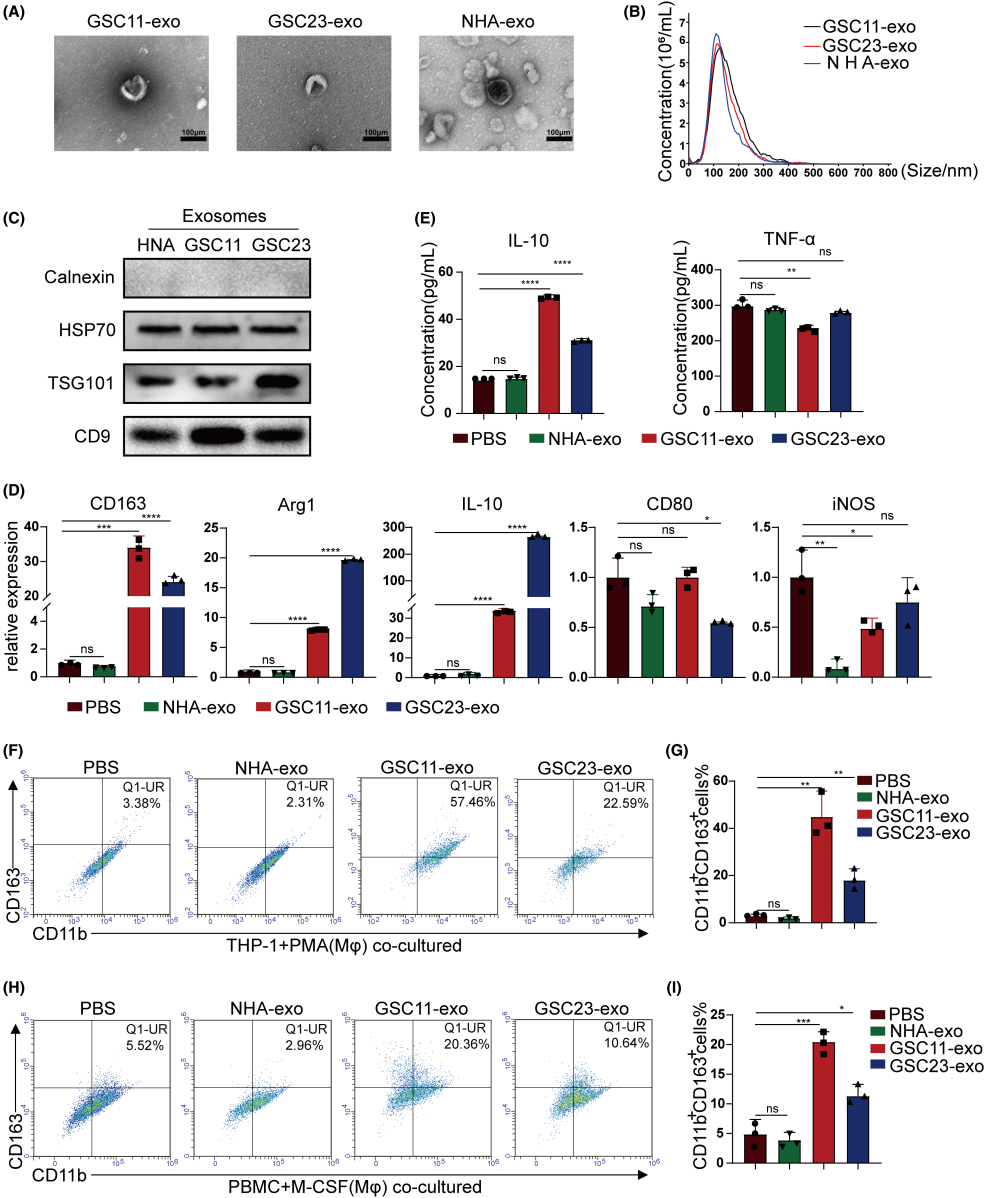
Glioblastoma stem cells (GSCs)‐exo induces M2 macrophage polarization. (A) Representative transmission electron microscopy (TEM) images of GSC11, GSC23, and NHA cell‐secreted exosomes (scale bar, 100 nm). (B) Nanoparticle tracking the size distribution of exosomes released by GSC11, GSC23, and NHA. (C) Western blot analysis was performed to detect typical exosomal biomarkers (HSP70, TSG101, CD9, and Calnexin) in exosomes derived from NHA, GSC11, and GSC23 cell lines. (D) RT‐qPCR was applied using primers for M2 markers (CD163, Arg1, and IL‐10) and M1 markers (CD80 and iNOS) in PMA‐pretreated THP‐1 cells treated with PBS, NHA‐exo, GSC11‐exo, GSC23‐exo. (E) Macrophages were treated with PBS, NHA‐exo, GSC11‐exo, GSC23‐exo, and the supernatants cultured for 48 h were examined to determine the secretion of IL‐10 and TNF‐α via ELISA. (F, G) Flow cytometry was used to examine the M2 macrophages (CD11b^+^CD163^+^) expression of PMA‐pretreated THP‐1 cells treated with PBS, NHA‐exo, GSC11‐exo, GSC23‐exo, and quantification was performed. (H, I) Flow cytometry was used to detect the M2 macrophages (CD11b^+^CD163^+^) expression of M‐CSF‐pretreated PBMC cells treated with PBS, NHA‐exo, GSC11‐exo, GSC23‐exo, and quantification was performed. Data depict the mean ± standard deviation and are representative of three independent experiments (*p < 0.05; **p < 0.01; ***p < 0.001; ****p < 0.0001).

The exosomes from GSCs or NHAs were isolated by ultracentrifugation. TEM verified that the harvested exosomes were vesicle‐like structures with size ranged from 30 to 100 nm (Figure 2A). Particle size analysis of the exosomes further confirmed that these particles distributed mainly varied in 30–150 nm (Figure 2B), which contained their specific markers (HSP70, TSG101, and CD9) but negative for endoplasmic reticulum marker Calnexin (Figure 2C). To evaluate whether macrophages can engulf GSCs‐derived exosomes, PKH26 (red fluorescence)‐labeled GSCs exosomes were cocultured with macrophages, which disclosed that the unstained macrophages internalized the PKH26‐labeled exosomes obviously under confocal microscopic observation (Figure S1H).

To verify whether GSCs‐exo can regulate M2 macrophage polarization, macrophages were treated with PBS, NHA‐exo, or GSCs‐exo, respectively. When compared with the control M0 macrophages treated with PBS, the expression of M2 markers significantly increased in the M0 treated with GSCs‐exo, while the expression of M1 markers reduced or remained unchanged. When compared with the macrophages treated by the NHA‐derived exosomes, the expression of relevant macrophage markers had no obvious changes (Figure 2D). Then, secretion of IL‐10 and TNF‐α in macrophages culture supernatant was analyzed with ELISA. Compared with addition of PBS or NHA‐exo, GSCs exosomes (GSC11‐exo or GSC23‐exo) significantly increased the IL‐10 secretion of macrophages, while markedly decreased TNF‐α secretion (GSC11‐exo) or remained changed (GSC23‐exo) (Figure 2E). Furthermore, expression of CD163 in macrophages treated with PBS, NHA‐exo, or GSCs‐exo was investigated by flow cytometry. Consistent with the RT‐qPCR results, GSCs‐exo significantly promoted CD163 expression in M0 macrphages (Figure 2F,G). A total of 7 days after isolation from healthy donor whole blood and in vitro cultured in RPMI 1640 culture medium, human PBMCs‐derived macrophages underwent morphological changes as well after addition of GSCs exosomes (Figure S1I). Flow cytometry analysis showed that CD163 expression in PBMCs‐induced macrophages treated with GSCs‐exo also increased (Figure 2H,I). In summary, these data indicated that GSCs can polarize macrophages toward M2 polarization through regulation by GSCs secreted exosomes.

### 3.3 MiR‐6733‐5p was highly expressed in GSCs and enhanced the self‐renewal and stemness of GSCs

**FIGURE 3:**
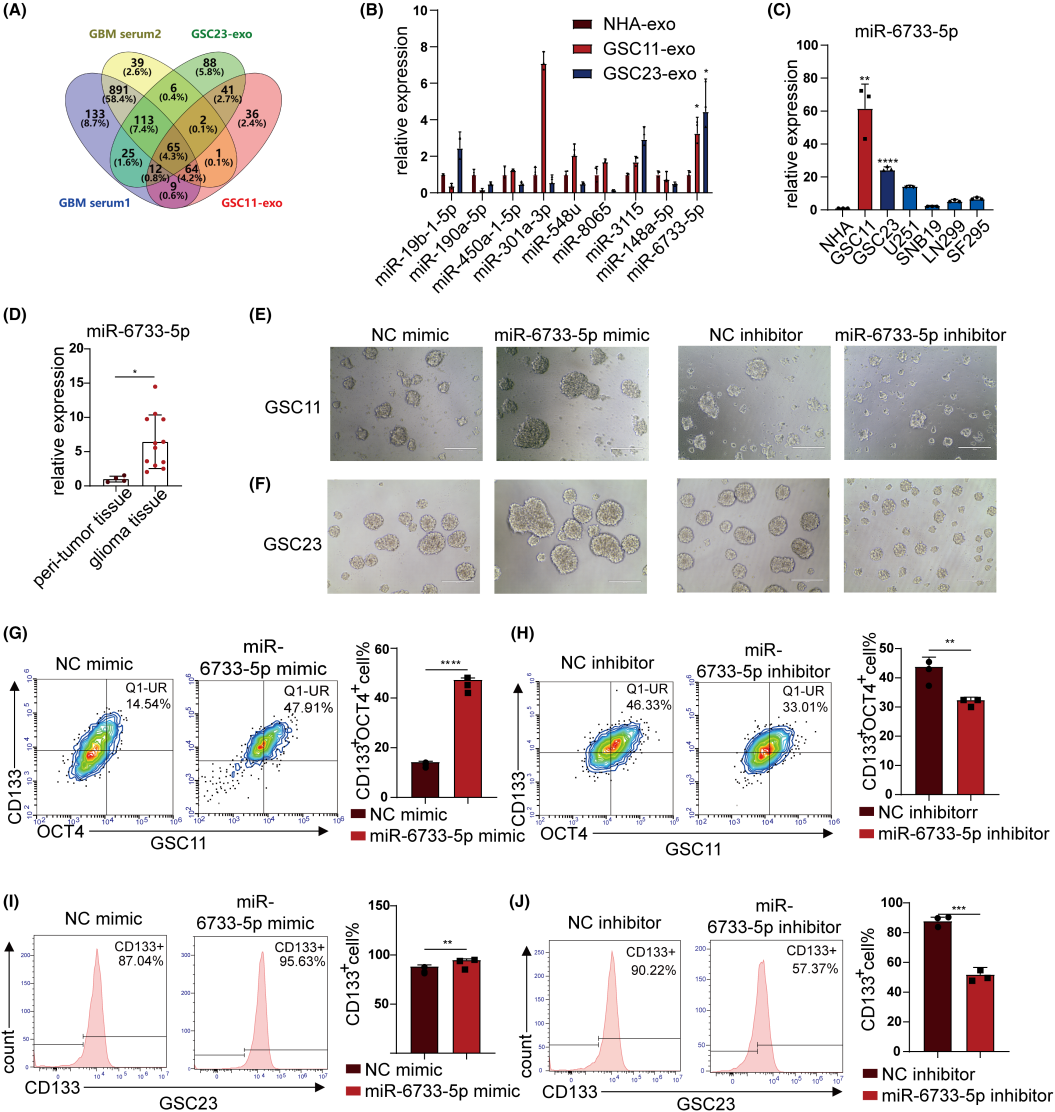
miR‐6733‐5p promoted the self‐renewal and stemness of Glioblastoma stem cells (GSCs). (A) Intersection of miRNA expression profile in four samples. miRNAs in serum of glioma patients (blue and yellow circle) and exosomal miRNAs enriched in GSC11 (red circle) and GSC23 (green circle) are shown. (B) Top 9 intersect miRNAs levels were analyzed by RT‐qPCR in NHA and GSCs (GSC11 and GSC23 cells)‐derived exosomes. (C) The expression levels of miR‐6733‐5p in GSCs and glioma cells were determined by RT‐qPCR. (D) The expression levels of miR‐6733‐5p in peri‐tumor tissue and glioma tissues were determined by RT‐qPCR. (E, F) Representative images showing neurosphere formation GSC11 and GSC23 treated with miR‐6733‐5p mimics/inhibitors (Scale bar, 200 μm). (G, H) Flow cytometry was applied to measure the surfaces marker (CD133) and intracellular marker (OCT4) of GSC11 treated with miR‐6733‐5p mimics/inhibitors and the ratio of CD133‐positive plus OCT4‐positive (CD133^+^OCT4^+^) GSC11 were quantitated. (I, J) Flow cytometry was applied to measure the surface marker (CD133) of GSC23 treated with miR‐6733‐5p mimics/inhibitors and the ratio of CD133‐positive (CD133^+^) GSC23 were quantitated. Data depict the mean ± standard deviation and are representative of three independent experiments (*p < 0.05; **p < 0.01; ***p < 0.001; ****p < 0.0001).

To reveal the potential exosomal miRNAs involved in GBM development, bioinformatic analysis on expression profiles of miRNAs in serum of GBM patients and healthy donors was predicted with the latest two miRNA‐Seq datasets from Gene Expression Omnibus database (GEO) (GSE139031, GSE113740). Besides, miRNA sequencing for exosomes derived from GSCs and NHAs was performed and the intersect miRNAs of the abovementioned four groups were shown in a Venn diagram (Figure 3A), and we finally chose miR‐6733‐5p for further study (Figure 3B). The RT‐qPCR results showed that miR‐6733‐5p was the highest expression in GSCs and higher than glioma cells (U251, LN229, SNB19, and SF295) (Figure 3C). Compared with the peri‐tumor tissue, miR‐6733‐5p level elevated obviously in the GBM tissue (Figure 3D), indicating that aberrantly high expression of miR‐6733‐5p may be associated with GBM development. Sphere‐formation assay showed that sphere‐forming ability significantly elevated after miR‐6733‐5p overexpression and decreased after miR‐6733‐5p knockdown both in GSC11 and GSC23 cells (Figure 3E,F). Flow cytometry analysis revealed that the CD133 and OCT4 expression significantly elevated after miR‐6733‐5p overexpression, and decreased after miR‐6733‐5p knockdown both in GSC11 and GSC23 cells (Figure 3G–J). These data suggested that miR‐6733‐5p was highly expressed in GSCs and enhanced the self‐renewal and stemness of GSCs.

### 3.4 MiR‐6733‐5p highly expressed in GSCs‐exo can be transferred to macrophages

**FIGURE 4:**
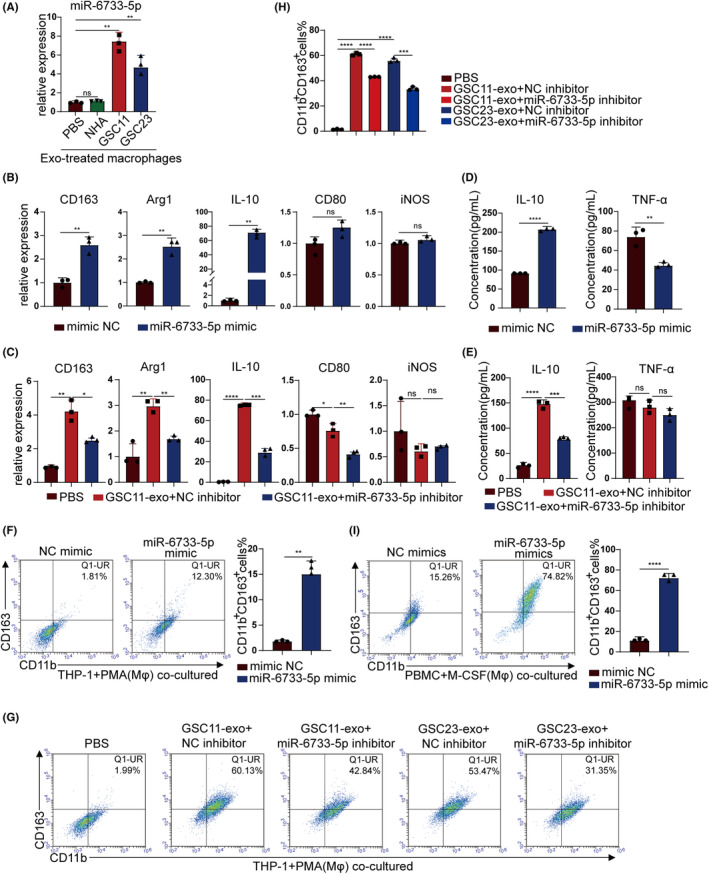
Glioblastoma stem cells (GSCs)‐derived exosomal miR‐6733‐5p induces M2 macrophage polarization. (A) Macrophages were incubated with GSC11‐exo or HNA‐exo for 48 h. The miR‐6733‐5p expression levels in macrophages were determined using RT‐qPCR. (B, C) THP1 (Mφ) cells transfected with mimics NC or miR‐6733‐5p mimics or incubated with GSC11‐exo and transfected with NC inhibitors or miR‐6733‐5p inhibitors. RT‐qPCR was adopted to detect the levels of M2 and M1 markers in THP1 (Mφ) cells. (D, E) Macrophage transfected with mimics NC or miR‐6733‐5p mimics or treated with GSC23‐exo and transfected with NC inhibitors or miR‐6733‐5p inhibitors, and the supernatants of 48‐h cultures were used to determine the secretion of IL‐10 and TNF‐α via ELISA. (F–I) Flow cytometry and quantification were performed to analyze the proportion of CD11b^+^CD163^+^ macrophages transfected with mimics NC or miR‐6733‐5p mimics or incubated with GSC11‐exo or GSC23‐exo and transfected with NC inhibitors or miR‐6733‐5p inhibitors. Data depict the mean ± standard deviation and are representative of three independent experiments (*p < 0.05; **p < 0.01; ***p < 0.001; ****p < 0.0001).

Mounting evidence indicated that miRNAs play a vital role in exosome mediate intercellular communications.^21^ In current studies, we disclosed that high level of miR‐6733‐5p in GSCs‐exo and can be engulf actively by macrophages. Macrophages incubated with GSCs‐exo expressed higher miR‐6733‐5p than those incubated with NHA exosomes or PBS only (Figure 4A). The role of miR‐6733‐5p on polarization of M2 macrophages was investigated, which showed that the expression of M2 markers increased after addition of miR‐6733‐5p mimics in macrophages derived from THP‐1 and decreased by miR‐6733‐5p inhibitors (Figure 4B,C; Figure S2A). ELISA results showed that miR‐6733‐5p mimics promoted the secretion of IL‐10 and inhibited the secretion of TNF‐α, while miR‐6733‐5p inhibitors decreased the secretion of IL‐10 without changing the secretion of TNF‐α (Figure 4D,E; Figure S2B). Flow cytometry analysis showed that the proportion of CD11b^+^CD163^+^ double positive THP‐1 derived macrophages increased by miR‐6733‐5p mimics and decreased by miR‐6733‐5p inhibitors (Figure 4F–H). Consistent with this finding, the proportion of CD11b^+^CD163^+^ double positive macrophages (PBMCs) also increased (Figure 4I). Taken together, these data suggest that GSCs‐derived exosomal miR‐6733‐5p contributed to M2 polarization of macrophages in vitro.

### 3.5 GSCs‐derived exosomal miR‐6733‐5p directly activated IGF2BP3 expression in macrophages

**FIGURE 5:**
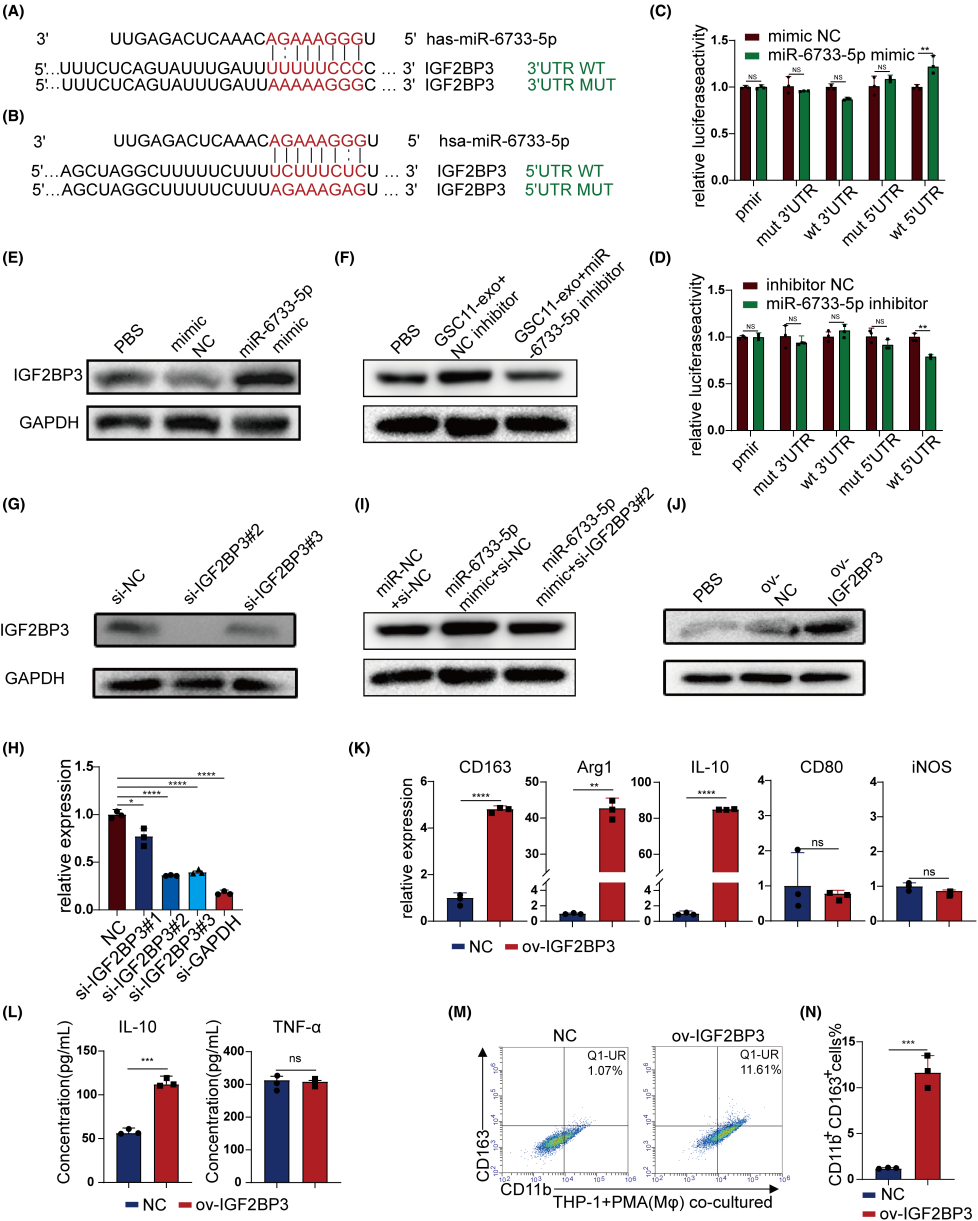
Glioblastoma stem cells (GSCs)‐derived exosomal miR‐6733‐5p directly targets IGF2BP3 in macrophages. (A, B) Schematic diagram of the wild‐type and mutated‐type binding site between miR‐6733‐5p and the IGF2BP3 3/5′UTR. (C, D) Luciferase reporter assays in HEK 293T cells with transfection of wild‐type or mutant IGF2BP3 3/5′‐UTR as well as either miR‐6733‐5p mimics or miR‐6733‐5p inhibitors were performed. Luciferase activity was normalized by the ratio of firefly and renilla luciferase signals and determined 48 h after transfection. (E–G) Comparison of IGF2BP3 expression in macrophages either treated with GSC11‐derived exosomes, or transfected with miR‐6733‐5p mimics/inhibitors, or transfected with si‐IGF2BP3, respectively, by Western blot. (H) The RT‐qPCR results showed that knocked down IGF2BP3 with small interfering RNAs significantly decreased the expression of IGF2BP3. (I, J) Comparison of IGF2BP3 expression in macrophages either transfected with miR‐6733‐5p mimics, or transfected with si‐IGF2BP3, or transfected with ov‐IGF2BP3, respectively, by Western blot. (K) Macrophages were transfected with plasmids overexpressing nonsense sequence or full‐length IGF2BP3 cDNA. RT‐qPCR was adopted to detect the levels of M2 (CD163, Arg1, and IL‐10) and M1 markers (CD80 and iNOS) in THP1 (Mφ) cells. (L) Macrophages were transfected with plasmids overexpressing nonsense sequence or full‐length IGF2BP3 cDNA. The supernatants of cultures were used to determine the secretion of IL‐10 and TNF‐α via ELISA. (M, N) Flow cytometry was applied to measure CD11b^+^CD163^+^ macrophages transfected with plasmids overexpressing nonsense sequence or full‐length IGF2BP3 cDNA, and quantification was performed. Data depict the mean ± standard deviation and are representative of three independent experiments (*p < 0.05; **p < 0.01; ***p < 0.001; ****p < 0.0001).

miRNAs can either target 3′UTR of mRNAs, resulting in repression of translation or degradation of mRNA targets, or target 5′UTR to induce activation of translation.^22^, ^23^, ^24^ To deeply explore the downstream target gene of GSCs‐derived exosomal miR‐6733‐5p in the induction of macrophage toward M2 polarization, we identified its candidate target gene IGF2BP3 (insulin‐like growth factor 2 mRNA binding protein 3) using online prediction tools (mirDIP). Besides, a search in the RNAhybird database and Mirmap database revealed two putative complementary binding sites on IGF2BP3 3′‐UTR and 5′‐UTR of miR‐6733‐5p (Figure 5A,B). To further validate whether miR‐6733‐5p directly targets IGFBP3, the 3′ or 5′‐UTR of IG3BP2 was constructed and cloned into the pmirGLO vector. Luciferase report assay disclosed miR‐6733‐5p mimics/inhibitors failed to change the luciferase activity of 3′‐UTR in HEK 293T cells (Figure 5C,D). However, luciferase activity of 5′‐UTR increased significantly after cotransfection of miR‐6733‐5p mimics, compared with the mutant vector. Conversely, miR‐6733‐5p inhibitors decreased the luciferase activity of 5′‐UTR and had a minimal effect on the mut group (Figure 5C,D). Furthermore, Western blot showed that exosomal miR‐6733‐5p increased IGF2BP3 expression in macrophages, while inhibition of miR‐6733‐5p decreased IGF2BP3 expression (Figure 5E,F; Figure S2C,D). Knocking down of IGF2BP3 with small interfering RNAs was performed, and knockdown efficiency in macrophages was verified by Western blot and RT‐qPCR (Figure 5G,H; Figure S2E). The effect of IGF2BP3 knockdown can be partly attenuated by transfection of miR‐6733‐5p mimics (Figure 5I; Figure S2F). These results implied that miR‐6733‐5p directly targeted IGF2BP3 and increased the expression of IGF2BP3.

### 3.6 GSCs exosomal miR‐6733‐5p targeted IGF2BP3 and induced M2 macrophages polarization by regulating AKT signaling pathway

**FIGURE 6:**
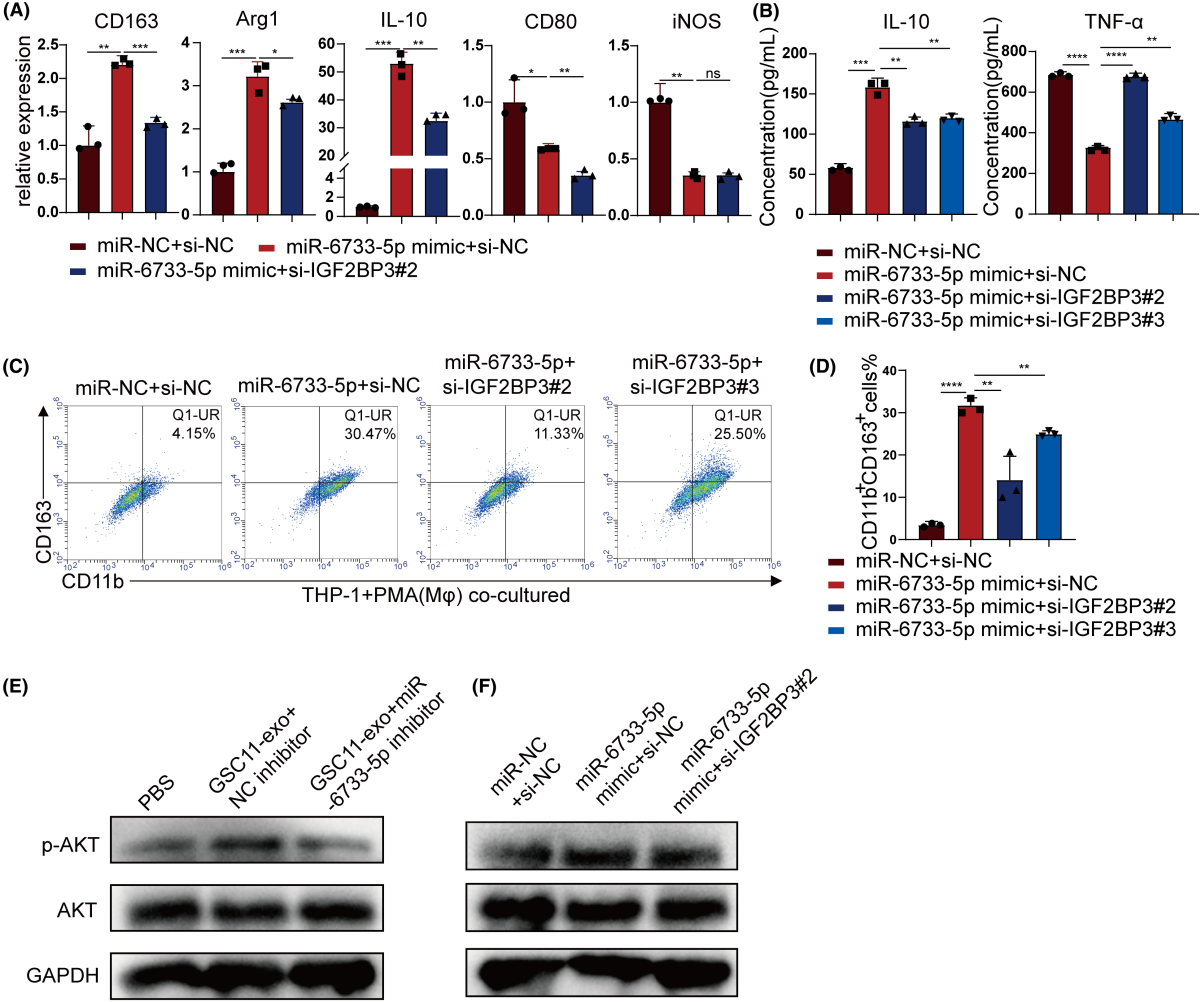
Glioblastoma stem cells (GSCs)‐derived exosomal miR‐6733‐5p targets IGF2BP3 to induce M2 polarization by activating the AKT pathway. (A) RT‐qPCR was performed M2 markers (CD163, Arg1, and IL‐10) and M1 markers (CD80 and iNOS) in PMA‐pretreated THP‐1 cells transfected with miR‐NC or miR‐1246 mimics and si‐NC or si‐IGF2BP3#2. (B) Macrophages were transfected with miR‐NC or miR‐1246 mimic and si‐NC or si‐IGF2BP3#2 or si‐IGF2BP3#3. The supernatants of cultures were used to determine the secretion of IL‐10 and TNF‐α via ELISA. (C, D) The macrophages were transfected with miR‐NC or miR‐1246 mimics and si‐NC or si‐IGF2BP3#2 or si‐IGF2BP3#3. The induced CD11b^+^CD163^+^ macrophages were determined by flow cytometry, and quantification was performed. (E, F) Western blot analysis of AKT pathway‐related proteins in THP‐1‐derived macrophages treated with miR‐NC or miR‐1246 mimics and si‐NC or si‐IGF2BP3#2. Data depict the mean ± standard deviation and are representative of three independent experiments (*p < 0.05; **p < 0.01; ***p < 0.001; ****p < 0.0001).

To explore the function of IGF2BP3 in macrophages, overexpression efficiency in macrophages was verified by Western blot (Figure 5I; Figure S2G). Overexpression of IGF2BP3 was conducted in THP‐1 cells‐derived macrophages, and RT‐qPCR showed that overexpression of IGF2BP3 significantly increased the expression of CD163, Arg1, and IL‐10 (Figure 5K), which was also verified with flow cytometry analysis and ELISA (Figure 5L–N). For evaluating the effect of targeting IGF2BP3 on miR‐6733‐5p induced M2 macrophage polarization, IGF2BP3 knockdown reversed the effect of miR‐6733‐5p on M2 macrophage polarization (Figure 6A–D), indicating that GSCs‐derived miR‐6733‐5p induced M2 macrophages polarization by targeting IGF2BP3.

For IGF2BP3 is an activated regulator of AKT pathway,^25^ and AKT signaling pathway is involved in M2 polarization,^26^ it is reasonable to be speculated that exosomal miR‐6733‐5p can activate AKT signaling pathway by regulating IGF2BP3 expression. Western blot results showed that the expression of phosphorylated AKT in macrophages treated with exosomes derived from GSC11 cells increased obviously, and can be abolished by miR‐6733‐5p inhibitors (Figure 6E; Figure S2H). In addition, the effects of IGF2BP3 knockdown can be partially attenuated by miR‐6733‐5p overexpression (Figure 6F; Figure S2I). Collectively, these results suggested that GSCs‐derived miR‐6733‐5p regulated IGF2BP3 expression to activate AKT signaling in macrophages, thus polarizing macrophages toward the M2 phenotype.

### 3.7 M2 macrophage polarization induced by GSCs‐derived exosomal miR‐6733‐5p promotes GBM growth

**FIGURE 7:**
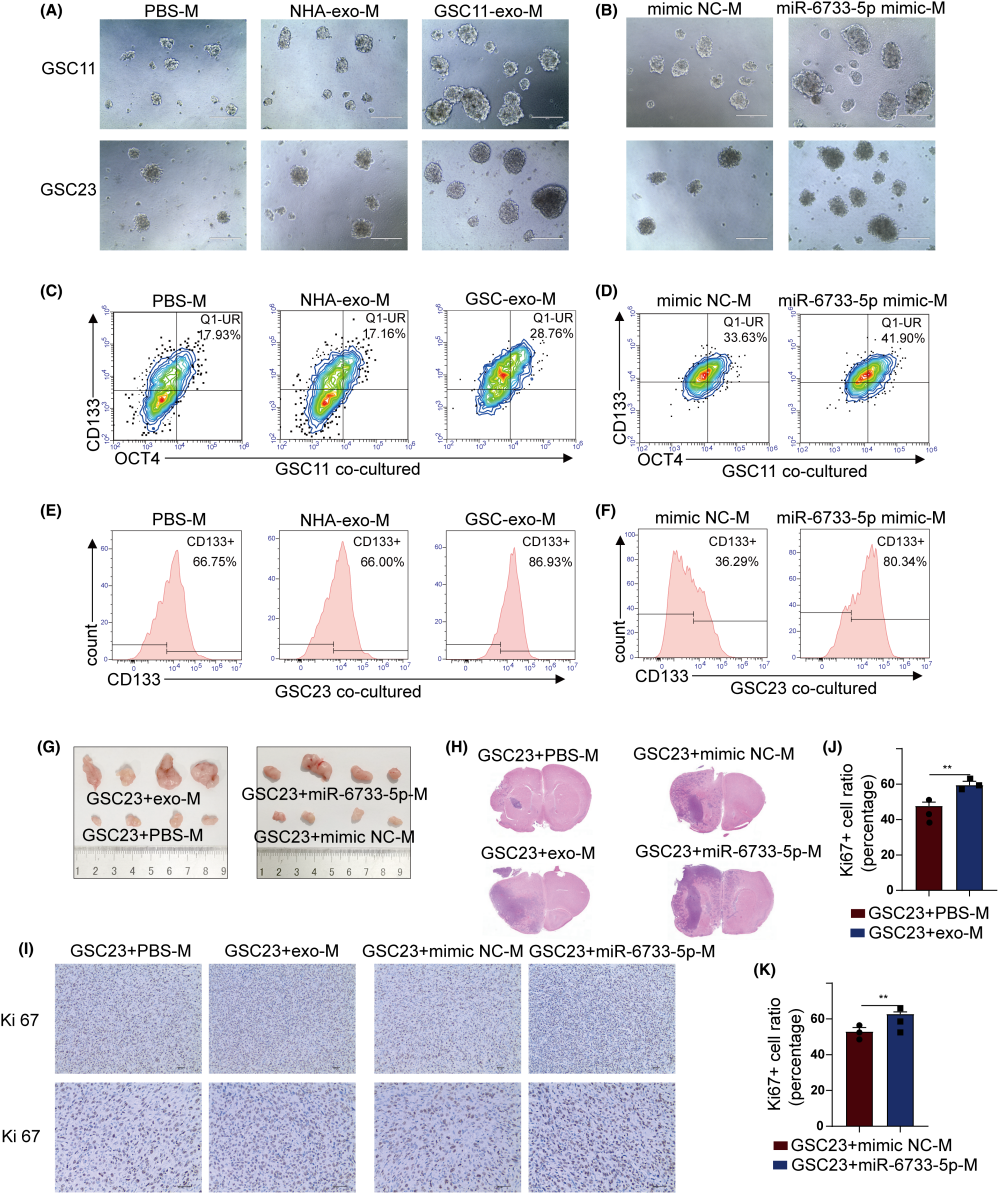
M2 macrophage induced by exosomal miR‐6733‐5p promote malignant behaviors of gliomas. (A, B) Representative images showing neurosphere formation GSC11/23 cocultured with THP1 (Mφ) treated with PBS, NHA‐exo, GSC11/23‐exo, and transfected with miR‐6733‐5p mimics (Scale bar, 200 μm). (C, D) Flow cytometry was applied to measure the surfaces marker (CD133) and intracellular marker (OCT4) of GSC11 cocultured with THP1 (Mφ) treated with PBS, NHA‐exo, and GSC11‐exo, and transfected with miR‐6733‐5p mimics. (E, F) Flow cytometry was applied to measure the surfaces marker (CD133) of GSC23 cocultured with THP1 (Mφ) treated with PBS, NHA‐exo, and GSC23‐exo and transfected with miR‐6733‐5p mimics. (G, H) In vivo evaluation of tumorigenesis in subcutaneous and orthotopic xenograft nude mice bearing GSC23 with THP1 (Mφ) treated with PBS or GSC23‐exo, and transfected with miR‐6733‐5p mimics. And representative tumor xenografts of HE staining images are shown. (I–K) Representative images and quantification of IHC staining for Ki67 in sections from the tumors (scale bar, 40 μm). Data depict the mean ± standard deviation and are representative of three independent experiments (**p < 0.01).

The role of M2 macrophages induced by GSCs‐derived exosomal miR‐6733‐5p on self‐renewal and stemness of GSCs was evaluated in an indirect coculture system. Sphere‐formation assay showed that both GSCs‐exo and miR‐6733‐5p mimics induced M2 macrophages significantly elevated sphere‐forming ability of GSCs (GSC11 and GSC23 cells), compared to the macrophages supplemented with PBS or NHA‐exo (Figure 7A,B). Flow cytometry analysis revealed that the proportion of CD133^+^OCT4^+^ double‐positive GSC11 cells increased, as well as CD133^+^ GSC23 cells, when cocultured with GSCs‐exo or miR‐6733‐5p mimics induced M2 macrophages (Figure 7C–F; Figure S3A–D). To validate these results in vivo, macrophages treated with GSCs‐exo or miR‐6733‐5p mimics and GSC23 cells were co‐implanted into nude mice to establish subcutaneous and orthotopic xenografts. Four weeks after implantation, the subcutaneous tumor volume and weight increased robustly in the mice with GSCs‐exo or miR‐6733‐5p mimics treated macrophages (Figure 7G; Figure S3E–H). HE and IHC staining showed that macrophages treated with GSCs‐exo or miR‐6733‐5p mimics resulted in more diverse aggressiveness and higher Ki67, F4/80, and IGF2BP3 expression of the tumors (Figure 7H–K; Figure S3I–K). In Addition, RT‐qPCR showed that macrophages treated with GSCs‐exo or miR‐6733‐5p mimics resulted in more expression of M2 markers (Figure S3L,M).

**FIGURE 8:**
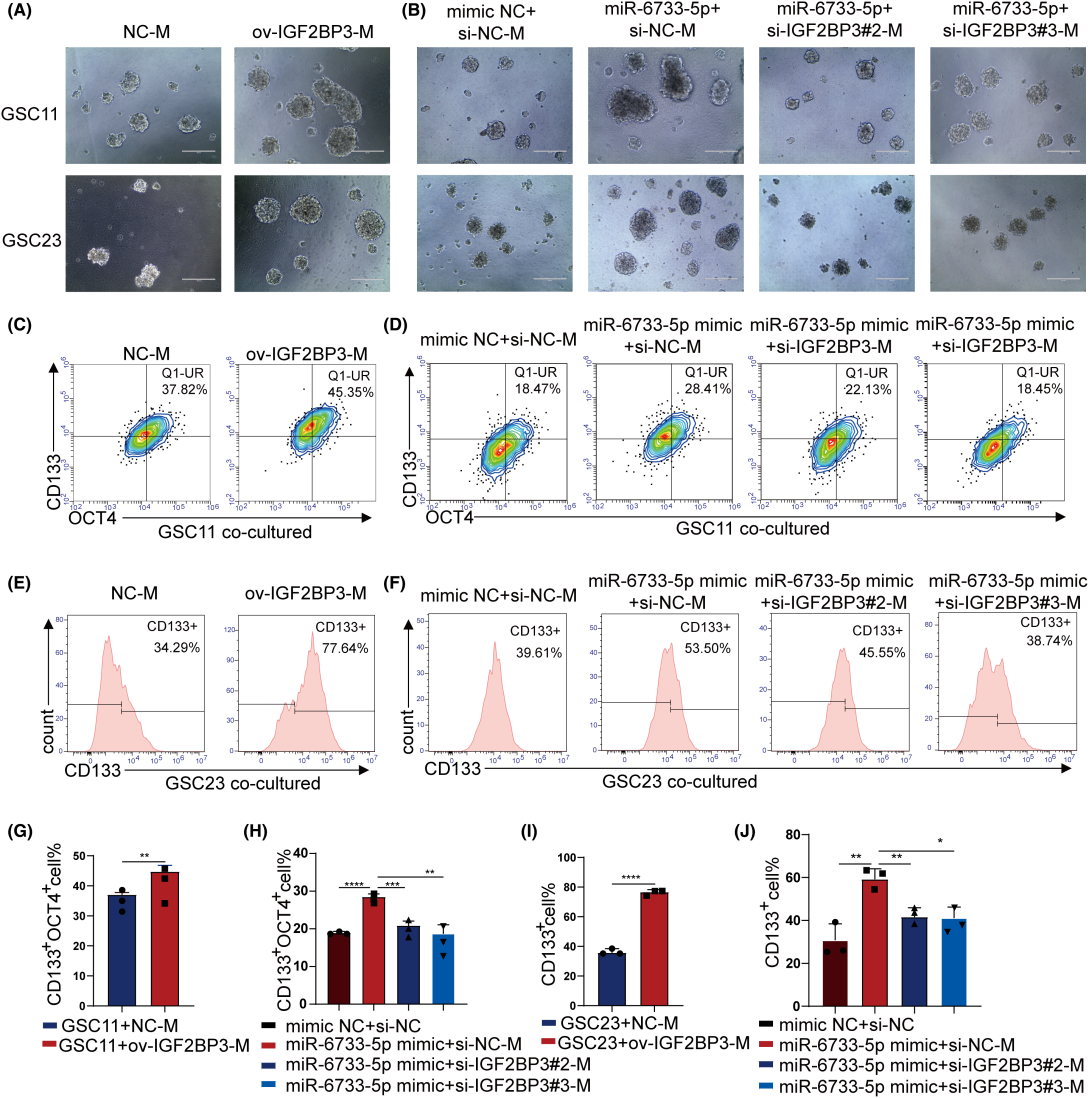
M2 macrophage induced by IGF2BP3 enhence the stemness of glioblastoma stem cells (GSCs). (A, B) Representative images showing neurosphere formation GSC11/23 cocultured with THP1 (Mφ) transfected with plasmids overexpressing nonsense sequence or full‐length IGF2BP3 cDNA and transfected with miR‐NC or miR‐1246 mimics and si‐NC, si‐IGF2BP3#2 or si‐IGF2BP3#3 (scale bar, 200 μm). (C, D) Flow cytometry were performed to analyze the proportion of CD133^+^OCT4^+^GSC11 cocultured with macrophages transfected with plasmids overexpressing nonsense sequence or full‐length IGF2BP3 cDNA and transfected with miR‐NC or miR‐1246 mimics and si‐NC, si‐IGF2BP3#2 or si‐IGF2BP3#3. (E–J) Flow cytometry was applied to measure the surfaces marker (CD133) of GSC23 cocultured with THP1 (Mφ) transfected with plasmids overexpressing nonsense sequence or full‐length IGF2BP3 cDNA and transfected with miR‐NC or miR‐1246 mimics and si‐NC, si‐IGF2BP3#2 or si‐IGF2BP3#3, and quantification was performed. Data depict the mean ± standard deviation and are representative of three independent experiments (*p < 0.05; **p < 0.01; ***p < 0.001; ****p < 0.0001).

To elucidate whether overexpressing IGF2BP3 in macrophages promote GSCs maintenance, the sphere‐formation assay and flow cytometry assay were performed, which demonstrated that macrophages overexpressing IGF2BP3 significantly increased the efficiency of sphere‐forming ability and stemness (Figure 8A,B). IGF2BP3 knockdown also reversed the effect of miR‐6733‐5p overexpressing macrophages on promoting GSCs maintenance (Figure 8C–J).

## 4 DISCUSSION

Considerable attention has been focused on the significance of tumor environment (TME) on tumor progression, a complex community that includes cancer cells, immune‐inflammatory cells, and diverse stromal cells and variety of regulating factors.^4^ Targeting M2 TAMs in TME is considered as an potential effective therapeutic strategy against cancer.^27^ Our previous studies have found that GBM could promote glioma‐associated macrophage infiltration and M2 polarization.^28^ Exosomes are membrane‐enclosed extracellular vesicles carrying biomolecules that include proteins and nucleic acids, especially miRNAs.^16^ In the current studies, we explored the effects and underlying mechanisms of cosstalk between GSCs and macrophages on M2 polarization, which disclosed that GSCs‐derived exosomal miR‐6733‐5p can induce M2 macrophage polarization, and polarized M2 macrophages promote self‐renew of GSCs inturn, which implied the mutual promotion of GSCs and TAMs on remodeling GBM microenvironment to favor GBM development.

Exosomes secreted by various tumor cells or stromal cells can influence tumor progression by transfer of bioactive molecules among different cell types in the TME.^29^, ^30^ Recently, accumulating studies have shown that tumor cells derived exosomes were involved in remodeling of immune suppressive microenvironment through crosstalk between tumor cells and tumor‐associated stromal cells.^15^ Tumor cells–derived exosomes can induce exhaustion of natural killer cells and induce resistance to immunocheckpoint blocking therapy.^31^, ^32^ Besides, tumor‐derived exosomes can also induce pro‐tumor activation of neutrophils to facilitate tumorigenesis.^33^, ^34^ Besides, our current studies demonstrated that GSCs can induce the polarization of macrophages toward M2 phenotypes via delivering GSCs exosomes.

MicroRNAs (miRNAs) are a class of small, noncoding RNAs that regulate the translation of target genes at posttranscription level. The abnormal expression of miRNAs and dysregulation of miRNA regulators are involved in the progression of many malignancies.^35^ The biological function of miR‐6733‐5p in tumor development has not yet been elucidated previously. Our investigations disclosed that miR‐6733‐5p was significantly enriched in GSCs, GSCs exosomes, and GBM tumor surgical specimen of patients, and further discovered that it can be delivered intracellularly via engulf of exosomes by macrophages. Besides, we demonstrated that intracellular miR‐6733‐5p can bind with IGF2BP3 to induce M2 macrophage polarization via regulating the AKT pathway.

IGF2BP3, together with IGF2BP1 and IGF2BP2, are members of RNA‐binding protein family that belongs to mRNA‐binding proteins, which influence the cytoplasmic fate of mRNAs through localization, stability, and translation.^36^, ^37^ Recent studies have showed that IGF2BP3 played key roles in GBM maintenance and promoting the emergence of M2‐subtype macrophages, highlighting its vital roles on development of gliomas.^38^, ^39^ However, the associated mechanisms of IGF2BP3 in gliomas have never been fully elucidated. Our data disclosed that miR‐6733‐5p can bind to the 5′‐UTR of IGF2BP3 and promote its expression, furthermore, overexpression of IGF2BP3, modulated by miR‐6733‐5p, resulted in activation of AKT signaling, indicating that miR‐6733‐5p played crucial roles on promoting M2 macrophage polarization by regulating the IGF2BP3/AKT pathway.

TAMs are responsible for various tumor‐promoting activities associated with GBM development, progression, and resistance to therapies.^40^, ^41^ In this investigation, we found that GSCs‐derived exosomal miR‐6733‐5p induced the alternative M2 macrophage activation, which help to promote the self‐renewal and stemness of GSCs both ex vivo and in vivo, indicating that exosomes are important regulators of GBM microenvironment remodeling and act as an important messenger that mediated the cross‐talk between GSCs and relevant stromal cells.

In summary, we demonstrated that GSCs‐derived exosomal miR‐6733‐5p can induce M2 macrophage polarization by activation of the IGF2BP3/AKT pathway, and remodeled macrophages can further promote the self‐renewal and stemness of GSCs. Our findings shed light on the mechanisms of GSCs exosomal miR‐6733‐5p in the development of GBM immuno‐microenvironment and highlight this pathway as a potential therapeutic target against gliomas. However, one common issue associated with immunodeficiency mouse models in glioma research fields is the lack of T lymphocytes, which limits the interpretation of immune related experimental data. Our experimental design can be improved if applying more powerful in vivo characterization methods to further elucidate the role of macrophages in GSCs‐microenvironment. Recent studies have already reported that molecular magnetic resonance imaging (mMRI) may be useful for enhancing the feasibility and accuracy of in vivo glioma studies.^42^, ^43^ Using molecular MRI could help visualize biological processes at the molecular level in immunodeficiency mouse models, thereby improving future studies.

## 5 CONCLUSIONS

**FIGURE 9:**
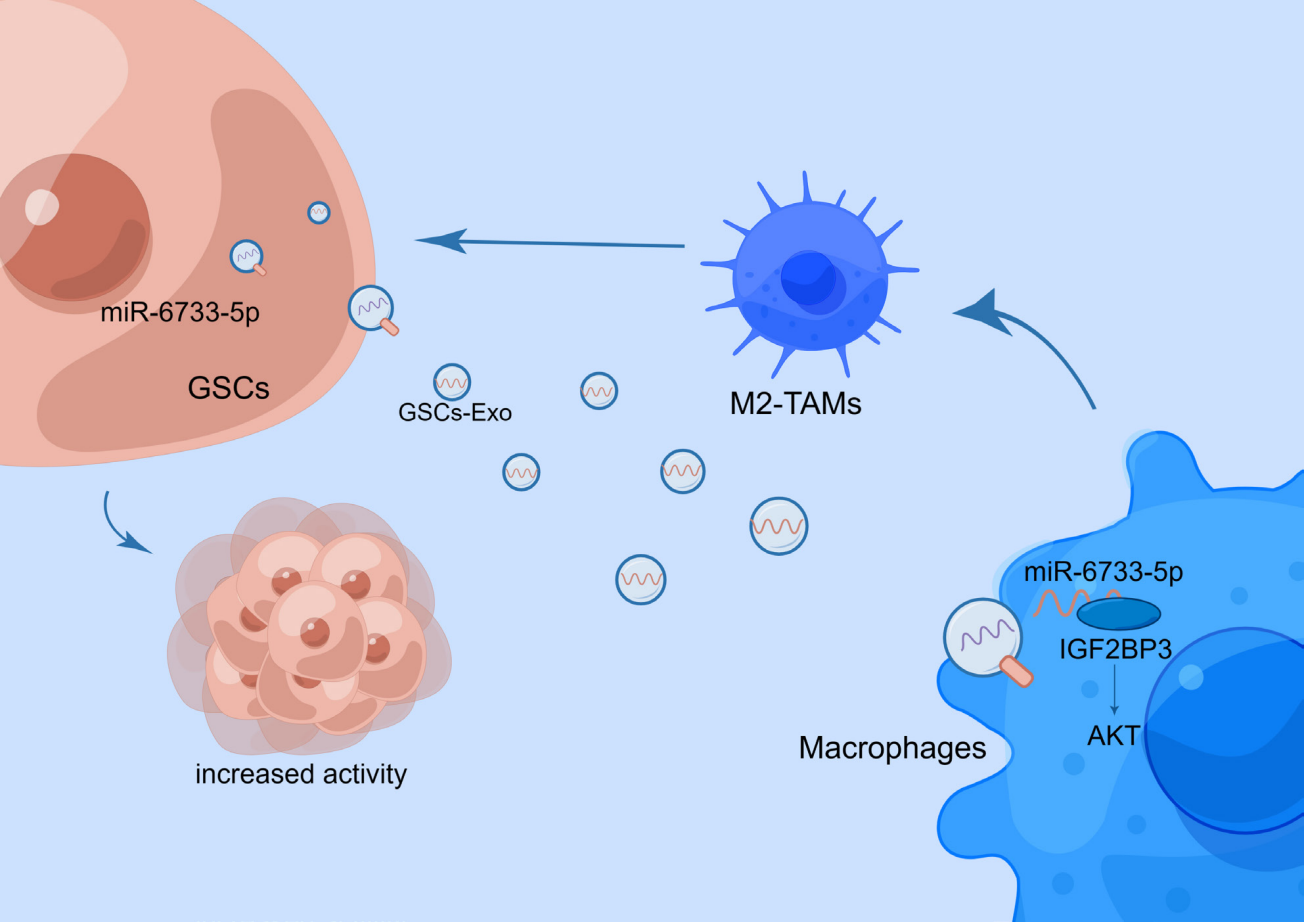
Molecular mechanism diagram by Figdraw. Glioblastoma stem cells (GSCs) exosomal miR‐6733‐5p induce macrophage M2 polarization of TAMs via targeting the IGF2BP3/AKT axis, which further facilitates the self‐renewal and stemness of GSCs.

We discovered that M2‐like TAMs infiltration and accumulation can be observed in GBM. GSCs can induce macrophage M2 polarization of TAMs, which further facilitates the self‐renewal and stemness of GSCs. Given that exosomes have been shown to transport miRNAs to alter cellular functions, we disclosed that miR‐6733‐5p was enriched in GSCs, GSCs‐derived exosomes, and surgical specimen of GBM patients. GSCs exosomal miR‐6733‐5p directly promotes the self‐renewal and stemness of GSCs, and induce macrophage M2 polarization of TAMs by positively targeting IGF2BP3 to activate the AKT signaling pathway, which further facilitates the self‐renewal and stemness of GSCs (Figure 9). These data provide a theoretical basis for the development of therapeutic strategy targeting exosomal miR‐6733‐5p in GBM treatment.

## AUTHOR CONTRIBUTIONS

SH, LL, and ZX visualization, writing original draft, investigation. AW, XL, XZ, and ZL formal analysis and data curation. SL, YL, and JY resources. SC and HL validation, software. JD supervision, project administration, draft review, editing, and funding acquisition.

## CONFLICT OF INTEREST STATEMENT

The authors have declared that no competing interest exists.

## Supporting information


Appendix S1

## Data Availability

All data used in this work can be acquired from the TCGA database (https://cancergenome.nih.gov/) and the GEO database (https://www.ncbi.nlm.nih.gov/geo/).
