# Supplementary material for: Exosomal miR‐6733‐5p mediates cross‐talk between glioblastoma stem cells and macrophages and promotes glioblastoma multiform progression synergistically
Source: CNS Neurosci Ther. 2023 Jun 12;29(12):3756–73. doi: 10.1111/cns.14296 (PMC10651992; doi:10.1111/cns.14296)
Supplement: Supplementary file 1 — Appendix S1 [file CNS-29-3756-s001.docx]

**Exosomal miR-6733-5p mediate cross-talk between GSC and macrophages synergistically promote GBM progress**

**Supplementary Material**

**Figure S1**

**
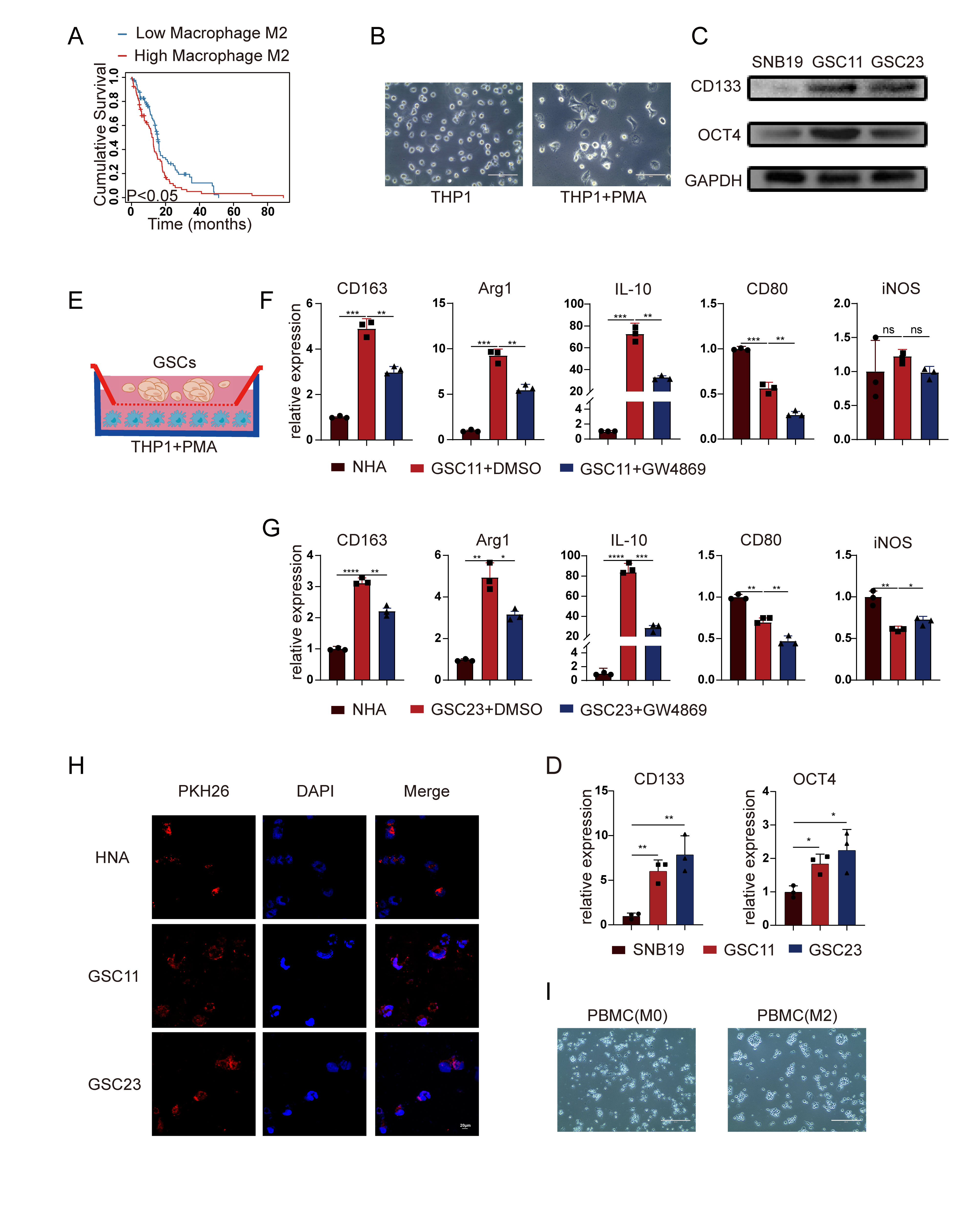
**

**GSCs induce macrophage towards M2 polarization.** (A) Kaplan-Meier survival plots of M2-like TAMs signature score showed a higher score indicated a poorer prognosis. P ˂ 0.05, log-rank test. (B) Human monocyte cell line THP-1 cells were incubated with PMA (100 ng/ml) for 24 h in vitro to induce them to differentiate into macrophages. (C-D) The key stemness biomarkers CD133 and OCT4 expression in SNB19, GSC11, and GSC23 cells was examined by western blot with quantification analysis. (E) Schematic illustration of the co-culturing model for THP1(Mφ) and GSCs(GSC11 or GSC23) using a transwell chamber (0.4-μm pore size). (F-G) THP1(Mφ) were co-cultured with GSCs treated with DMSO or exosome secretion inhibitor GW4869. qRT-PCR was adopted to detect the levels of M2 markers (CD163, Arg1 and IL10) and M1 markers (CD80 and iNOS). (H)Confocal microscopy images illustrated the process of PKH26-labeled exosomes transmitted to macrophages (Scale bar: 20μm). (I)Human peripheral blood mononuclear cells (PBMCs)-derived macrophages were morphological changed via treated GSCs-exo. Data depicts the mean ± standard deviation and are representative of three independent experiments. (*p < 0.05; **p < 0.01; ***p < 0.001;****p< 0.0001).

**Figure S2**

**
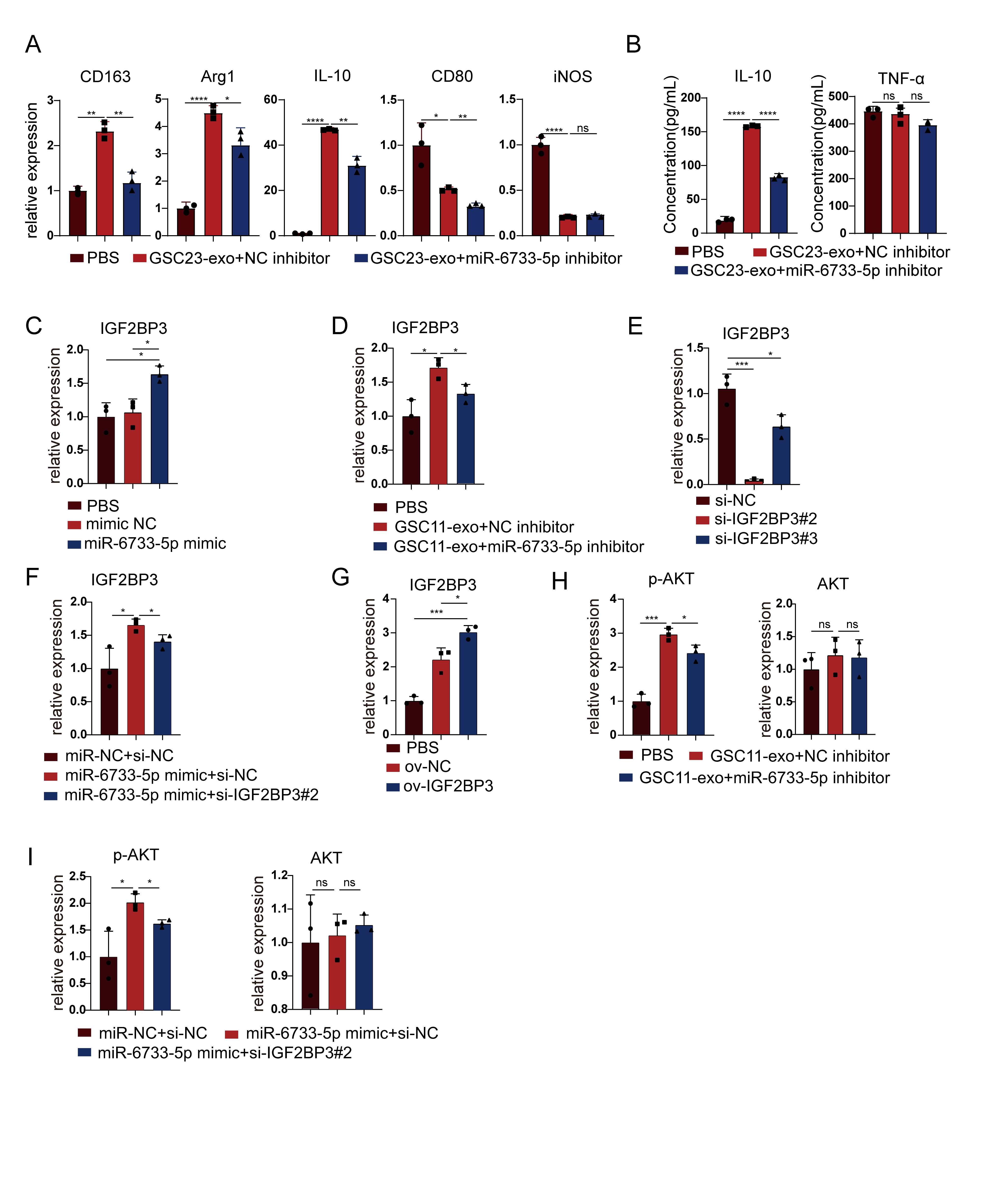
**

**miR-6733-5p inhibit M2 macrophage polarization induced by GSCs-exo.** (A) THP1(Mφ) cells incubated with GSC23-exo and transfected with NC inhibitors or miR-6733-5p inhibitors. RT-qPCR was adopted to detect the levels of M2 and M1 markers in THP1(Mφ) cells. (B) Macrophages transfected with mimic NC or miR-6733-5p mimic or treated with GSC23-exo and transfected with NC inhibitors or miR-6733-5p inhibitors, and the supernatants of 48-hour cultures were used to determine the secretion of IL-10 and TNF-α via ELISA. (C-I) Comparison of IGF2BP3, p-AKT, and AKT expression in macrophages either treated with GSC11-derived exosomes, or transfected with miR-6733-5p mimics/inhibitors, or transfected with si-IGF2BP3, or transfected with ov-IGF2BP3, respectively by western blot with quantification analysis. Data depicts the mean ± standard deviation and are representative of three independent experiments. (*p < 0.05; **p < 0.01; ***p < 0.001;****p< 0.0001)

**Figure S3**

**
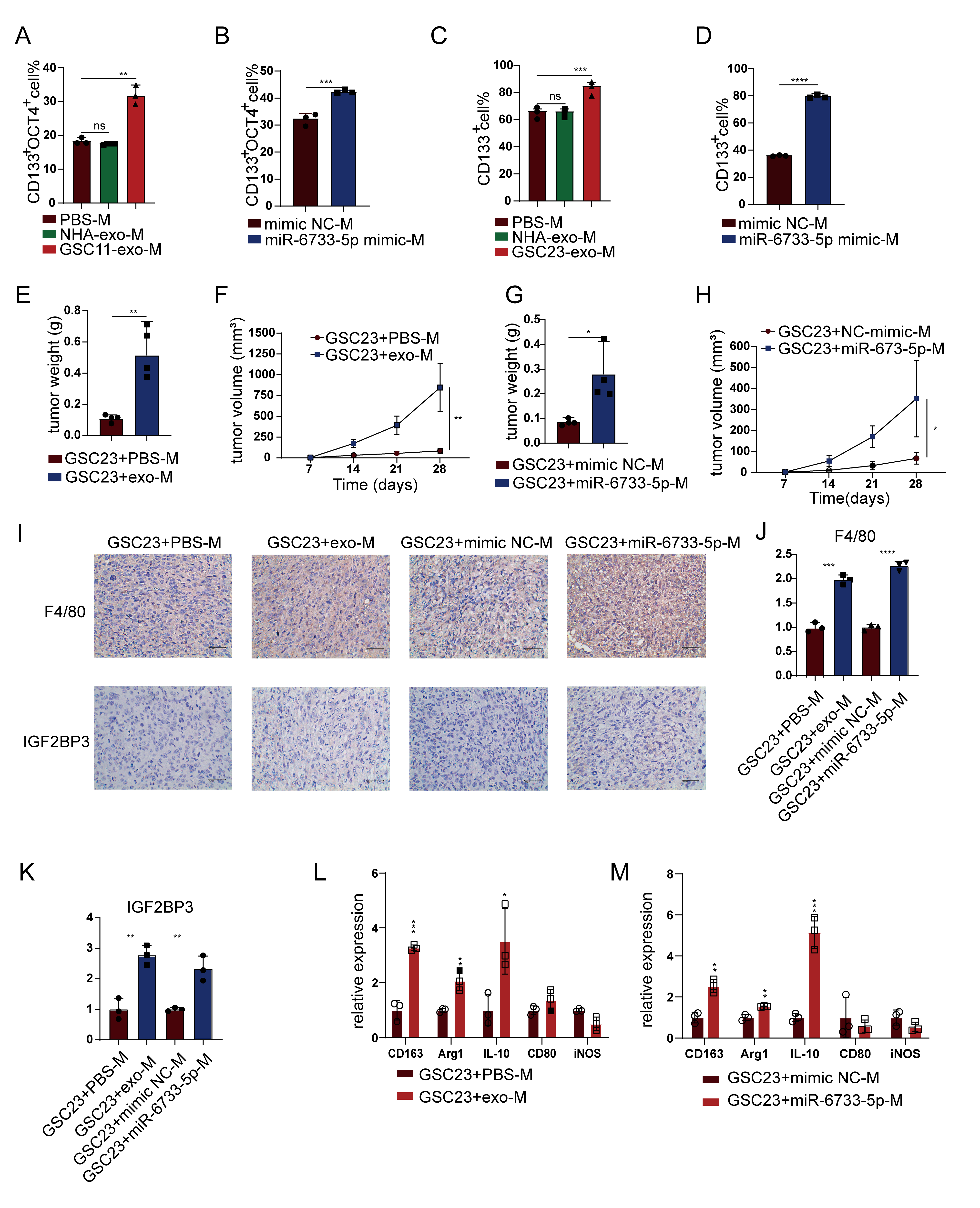
**

**M2 macrophage induced by exosomal miR-6733-5p promote malignant behaviors of gliomas.** (A-D) Flow cytometry was applied to measure CD133/OCT4 of GSC11 and CD133 of GSC23 cocultured with THP1(Mφ) treated with PBS, NHA-exo, and GSC11-exo, and transfected with miR-6733-5p mimics. The ratio of CD133-positive plus OCT4-positive (CD133^+^OCT4^+^) GSC11 and CD133-positive (CD133^+^) GSC23 were quantitated. (E-H) In vivo evaluation of tumorigenesis on volumes and weights of subcutaneous tumor in xenograft nude mice bearing GSC23 and THP1(Mφ) incubated with GSCs-exo or miR-6733-5p mimics. (I-K) Representative images of IHC staining for F4/80 and IGF2BP3 in sections from the intracerebral GSC23-transplantable tumors with quantification analysis. (scale bar, 40 µm). (L-M) RT-qPCR was adopted to detect the levels of M2 and M1 markers in tumors. Data depicts the mean ± standard deviation and are representative of three independent experiments. (*p < 0.05; **p < 0.01; ***p < 0.001; ****p< 0.0001).

**Table S1** Primers for RT-qPCR

| GAPDH forward: 5′- CAGGAGGCATTGCTGATGAT -3′ |
| --- |
| GAPDH reverse: 5′- GAAGGCTGGGGCTCATTT -3′ |
| CD68 forward: 5′- GGAAATGCCACGGTTCATCCA-3′ |
| CD68 reverse: 5′- TGGGGTTCAGTACAGAGATGC -3′ |
| CD11b forward: 5′- CTGTTTACCTGTTTCACGGAAC-3′ |
| CD11b reverse: 5′-GATTGCCTTGACTCTCAGTACT-3′ |
| IL-10 forward: 5′-GTTGTTAAAGGAGTCCTTGCTG-3′ |
| IL-10 reverse: 5′-TTCACAGGGAAGAAATCGATGA-3′ |
| CD163 forward: 5′-ATCAACCCTGCATCTTTAGACA-3′ |
| CD163 reverse: 5′- CTTGTTGTCACATGTGATCCAG-3′ |
| Arg1 forward: 5′- GGACCTGCCCTTTGCTGACATC-3′ |
| Arg1 reverse: 5′- TCTTCTTGACTTCTGCCACCTTGC-3′ |
| iNOS forward: 5′- GACTTTCCAAGACACACTTCAC-3′ |
| iNOS reverse: 5′- TTCGATAGCTTGAGGTAGAAGC-3′ |
| CD80 forward: 5′- GTGGTCACAATGTTTCTGTTGA -3′ |
| CD80 reverse: 5′- GTTCTTGTACTCGGGCCATATA -3′ |
| hsa-mir-6733-5p forward: 5′- AATCCGGATGGGAAAGACAAAC -3′ |
| hsa-mir-6733-5p reverse: 5′- CAGTGCAGGGTCCGAGGT-3′  hsa-mir-6733-5p RT：GTCGTATCCAGTGCAGGGTCCGAGGTATTCGCACTGGATACGACAACTCT |
| U6 forward: 5′- AGAGAAGATTAGCATGGCCCCTG -3′ |
| U6 reverse: 5′- ATCCAGTGCAGGGTCCGAGG -3′  U6 RT：  GTCGTATCCAGTGCAGGGTCCGAGGTATTCGCACTGGATACGACAAAATA |

Table S2. Sequences for siRNAs, microRNA mimics and inhibitors.

| si- IGF2BP3 #1: sense 5′- CCUUGAAAGUAGCCUAUAUTT-3′  antisense 5′- AUAUAGGCUACUUUCAAGGTT -3′ |
| --- |
| si- IGF2BP3 #2: sense 5′- GCAGGAAUUGACGCUGUAUTT-3′  antisense 5′- AUACAGCGUCAAUUCCUGCTT -3′ |
| si- IGF2BP3 #3: sense 5′- GCUGCUGAGAAGUCGAUUATT-3′  antisense 5′- UAAUCGACUUCUCAGCAGCTT -3′ |
| miR-6733-5p mimics: sense 5′- UGGGAAAGACAAACUCAGAGUU-3′  antisense 5′-CUCUGAGUUUGUCUUUCCCAUU-3′ |
| miR-6733-5p inhibitor: sense 5′-AACUCUGAGUUUGUCUUUCCCA-3′ |
